# Relevance of Rabbit VX2 Tumor Model for Studies on Human Hepatocellular Carcinoma: A MicroRNA-Based Study

**DOI:** 10.3390/jcm4121954

**Published:** 2015-12-04

**Authors:** Rajagopal N. Aravalli, Erik N. K. Cressman

**Affiliations:** 1 Department of Radiology, University of Minnesota Medical School, Minneapolis, MN 55455, USA; 2 Department of Interventional Radiology, M.D. Anderson Cancer Center, Houston, TX 77030, USA; ECressman@mdanderson.org

**Keywords:** hepatocellular carcinoma, VX2 tumor, microRNA, animal model, biomarker

## Abstract

MicroRNAs are small (~22 nt), noncoding RNA molecules that have critical cellular functions in proliferation, differentiation, angiogenesis and apoptosis. miRNA expression profiling has been used to create signatures of solid tumors and, in many cases, it has been shown to correlate with the severity of the disease. The rabbit VX2 tumor model has been used widely to study a number of human cancers. Our objective in this study is to generate an miRNA signature of the VX2 tumor and to identify miRNAs that are highly expressed in this aggressive tumor. In this study, we performed miRNA profiling of the rabbit VX2 tumor using a microarray that has probes for 1292 unique miRNAs. Their expression in tumor samples was quantified and analyzed. We found that 35 miRNAs were significantly up-regulated in the VX2 tumor. Among these, 13 human miRNAs and eight members of the *let-7* family were previously identified in cancers. In addition, we show that the expression of three miRNAs (miR-923, miR-1275, and miR-1308) is novel for the rabbit VX2 tumor, and their expression was not previously shown to be associated with any type of cancer. For the first time, we show the miRNA signature profile for a solid tumor in a rabbit model. miRNAs highly expressed in the VX2 tumor may serve as novel candidates for molecular biomarkers and as potential drug targets.

## 1. Introduction

Interest in image-guided interventions, particularly in oncology therapy, is continuously increasing. Ablation therapies for solid tumors abound and there, unfortunately, are no good, readily available large animal models for use in studying these treatments [1]. Rabbit VX2 tumors have been used extensively for evaluation of a number of these interventions, especially radiofrequency ablation and various transarterial embolization methods. It would seem prudent to have a reasonable understanding of the characteristics of the tumor and its relationship to the tumor being modeled, yet the literature on the molecular biology of the liver model of VX2 is sparse to nearly nonexistent [2]. While this may be ascribed to a number of factors, perhaps the single biggest reason is that the cell line has proven very difficult if not impossible to grow *in vitro* [3]. Over the past decade, microRNA (miRNA) technology has gained a great deal of attention for a number of reasons, including ease of analysis, specificity of results, and clinical implications in imaging and treatment because the expression of different miRNAs was found to be altered in a number of human cancers [4]. Fresh or frozen tissues can provide adequate material, and, therefore, it seems both appropriate and timely to study the profile of VX2 for subsequent comparison as data become available in the literature.

MicroRNAs (miRNA) are a class of short, non-coding cellular RNAs that regulate post-transcriptional gene expression of messenger RNAs. They are typically between 19 and 25 nucleotides in length and are generated from long endogenous transcripts [5]. To date, over 1000 miRNAs have been identified in mammalian cells and their number is growing exponentially. It is estimated that approximately 30% of all cellular mRNAs are regulated by miRNAs. Extensive studies during the past decade have revealed that miRNAs are involved in diverse cellular processes such as cell division, cellular differentiation, apoptosis, and organ development [6]. Such miRNAs serve as “signature sequences” for a given type of cancer. In addition, some miRNAs also function as either suppressors or inducers of cancer [7].

Animal models of human disease have been instrumental in enhancing our knowledge regarding biochemical, molecular and cellular events that take place in cells and also in drug development and metabolism. With advances in novel “omics-based” technologies and the advent of miRNA profiling, the focus has shifted towards the analysis of patient samples. It is now possible to evaluate the relevance of animal models for certain studies using these novel technologies. An example would be comparing the miRNA profiles of the animal models to those obtained with patient samples.

The rabbit VX2 carcinoma model was developed by Rous *et al.* [8,9]. While it was originally established from skin cancer of cottontail rabbits, it was subsequently shown to be transplantable in all strains of domestic rabbits [10]. Ever since the VX2 tumor was found to be caused by the infection with a papillomavirus [11], termed “cottontail rabbit papillomavirus (CRPV)”, it has been used widely as a model system to study a number of solid human cancers, including those of lung, bladder, neck, breast, liver and kidney [12,13,14,15,16,17]. Because of our interest in liver cancer, we were interested to compare the miRNA profile of VX2 with profiles from various human cancers already reported in the literature. Profiling of animal models will be invaluable as a method to help to determine whether or not they serve as relevant animal models of human tumors.

## 2. Experimental Section

### 2.1. Animals and Tumor Induction

Animal studies were carried out according to institutional guidelines. The VX2 tumor strain was implanted into New Zealand white rabbits as described previously [18] to generate cancer in the liver tissue, and tumor was propagated for 14 days prior to sacrifice. Tumor tissue was then harvested and placed in Hanks’ buffered salt solution (HBSS). Samples were then sliced into 1 mm^3^ cubes and transferred to cryotubes containing 0.5 mL freezing solution (70% FBS, 25% RPMI Medium 1640, 5% DMSO). Cryotubes were stored at −70 °C in a freezing container with 70% isopropyl alcohol until further use. The animal protocols used in this work were evaluated and approved by the Institutional Animal Care and Use Committee of the University of Minnesota.

### 2.2. RNA Isolation and Microarrays

One gram of the tumor tissue was used to isolate total RNA using a mirVana Isolation kit (Ambion, Austin, TX, USA). The small RNAs (<200 nt) were size fractionated and 3′-extended with a poly(A) tail using poly(A) polymerase. Microarrays were assayed using a service provider (LC Sciences, Houston, TX, USA). Total RNA was probed in triplicate on microarrays and data was analyzed by normalizing it with control probes. 4 μg of total RNA labeled with Cy3 was used to probe each microarray. Hybridization was performed overnight on a μParaFlo^®^ microfluidic chip using a micro-circulation pump (Atactic Technologies, Houston, TX, USA). Hybridizations used 6 × SSPE buffer (0.90 M NaCl, 60 mM Na_2_HPO_4_, 6 mM EDTA, pH 6.8) containing 25% formamide at 34 °C. After hybridization, signals were detected using fluorescence labeling with Cy3 (Invitrogen, Carlsbad, CA, USA). Hybridization images were collected using the GenePix 4000B laser scanner (Molecular Device, Sunnyvale, CA, USA) and quantified.

A human/mouse/rat microRNA microarray (MRA-1030) (LC Sciences) containing 1292 unique detection spots of a chemically modified nucleotide coding segment complementary to the target mature miRNAs as well as multiple positive controls was used in this study. Detection probes were prepared by *in situ* synthesis using the photogenerated reagent chemistry and melting temperatures for hybridization were balanced by chemical modifications of the detection probes. The probe content on microarrays was based on the miRBase version 21 (http://www.mirbase.org).

### 2.3. Data Analysis

Data were analyzed by first subtracting the background and were normalized using a cyclic LOWESS filter (locally-weighted regression) [19]. Data classification involved a hierarchical clustering method of average linkage and Euclidean distance metric, and was visualized with the Multiple Experimental Viewer (MeV) software (J. Craig Venter Institute, Rockville, MD, USA).

## 3. Results and Discussion

In this investigation, microRNA profiling was performed using a microarray chip that had probes for 1292 unique mature miRNA from human, mouse and rat species as no rabbit arrays are currently available. The partitioning of the array was as follows: 837 human probes, 599 mouse probes, and 350 rat probes. The expression levels of each miRNA were normalized to the positive control probe after deducting the background signal. The relative expression levels were then calculated using the MeV software. We found that the expression of 35 miRNAs was significantly elevated in the VX2 tumor. Among these, rat miRNAs rno-miR-200b, rno-miR-200c, and rno-miR-214 were homologs of human hsa-miR-200b and hsa-miR-200c, and hsa-miR-214, respectively. Interestingly, these are the only miRNAs from rat species that were up-regulated. Similarly, the expression of only two miRNAs from the mouse species, mmu-miR-705 and mmu-miR-762, was elevated. In contrast, overexpression of 31 human miRNAs was detectable in VX2 tumor samples. While the expression of some of these miRNAs was previously reported to be altered in human cancers, up-regulation of other miRNAs in tumors was not known.

**Figure 1:**
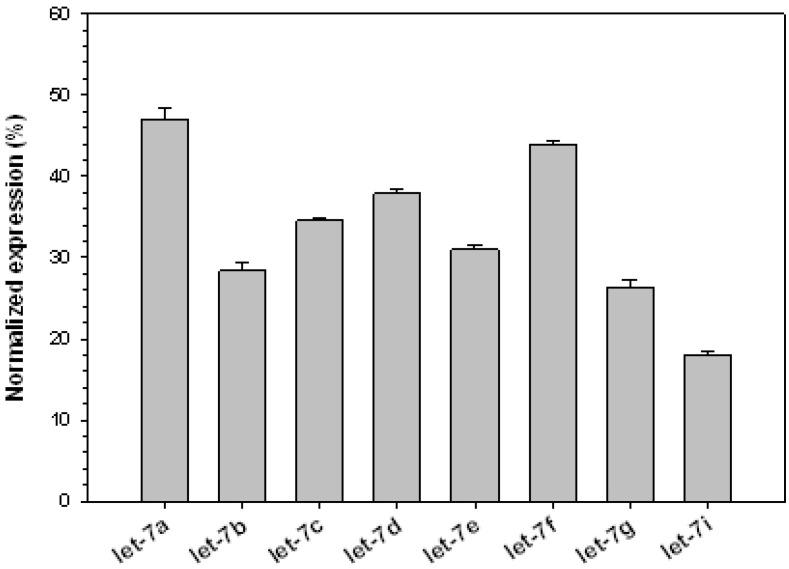
Expression of *let-7* family of miRNAs in rabbit VX tumors. miRNA microarray contained 17 sequences (including variants) of human *let-7* family members. Of these, only eight were up-regulated in the tumor samples used in this study. Signal intensities of each miRNA were calculated after normalizing the expression levels with positive controls. Data are presented as mean ± SD of triplicate samples.

*let-7* was the first identified human miRNA [20] and its family members are conserved across species. Misregulation of *let-7* family of miRNAs was reported in human cancers, suggesting that *let-7* may function as a tumor suppressor [21]. In the rabbit VX2 tumor, we have identified the up-regulation of eight members of the *let-7* family (Figure 1).

**Figure 2:**
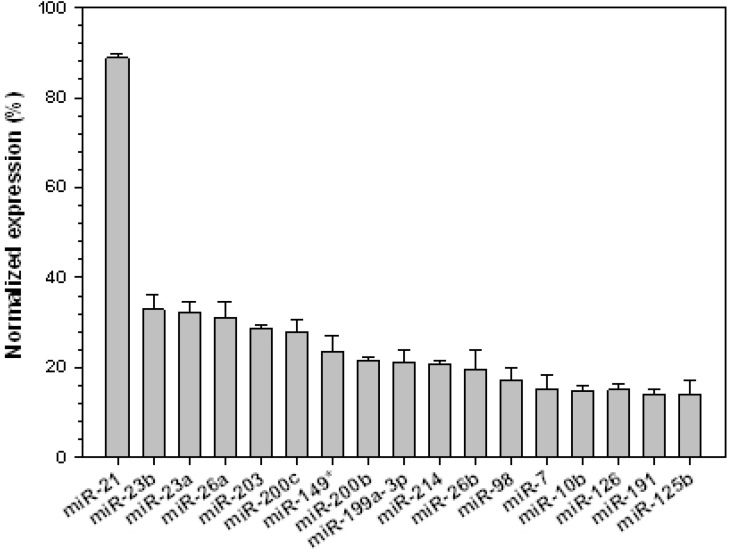
MicroRNAs upregulated in rabbit VX2 tumors that have been implicated previously in various human cancers. Signal intensities of each miRNA were calculated after normalizing the expression levels with positive controls. Data are presented as mean ± SD of triplicate samples.

In this study, the highest expression level was obtained for miR-21 (Figure 2). It is one of the most extensively studied miRNAs. Its expression is elevated in a number of cancers [22]. miR-21 regulates the tumor suppressor genes PTEN and tropomyosin [23,24] and promotes cellular transformation by mediating the downregulation of the programmed cell death 4 gene [25,26]. Knockdown of miR-21 was found to disrupt cancer growth in the brain [27].

Several other miRNAs were previously identified in human cancers (Figure 2). Among these, miRNA-98 is frequently up-regulated in the head and neck squamous cell carcinoma, where it targets the high mobility group A2 protein [28]. miR-221 regulates the expression of CDKN1C/p57 and CDKN1B/p27 in human hepatocellular carcinoma (HCC) [29]. In colon cancer, miR-126 inhibits the growth of neoplastic cells by targeting the cellular PI-3-kinase signaling pathway [30]. In human ovarian cancer, miR-214 promotes cell survival by inhibiting PTEN [31]. miR-26b, miR-23b, miR-203, and miR-23a were significantly up-regulated in bladder cancers [32]. Similarly, miRNAs that were up-regulated in other cancers such as miR-126 in colon cancer [30], miR-23a and miR-125b in HCC [33,34], miR-191 and miR-199a in myeloid leukemia [35], miR-200b [36], miR-10b and miR-26b in breast cancer [37,38], and miR-98 [28] in head and neck squamous cell carcinoma were also up-regulated in rabbit VX2 tumors. miR-200c [39], miR-203 [40], miR-23b [41], and miR-7 [42] were shown to be down-regulated in other cancers. Interestingly, however, they are up-regulated in the VX2 tumor. miR-126 was previously implicated in the suppression of cellular proliferation. It functions by inhibiting the expression of IRS-1 [43]. It was reported that miR-224 is significantly induced in HCC where it suppresses the activity of apoptosis inhibitor 5 [44]. However, we did not detect any up-regulation of miR-224.

**Figure 3:**
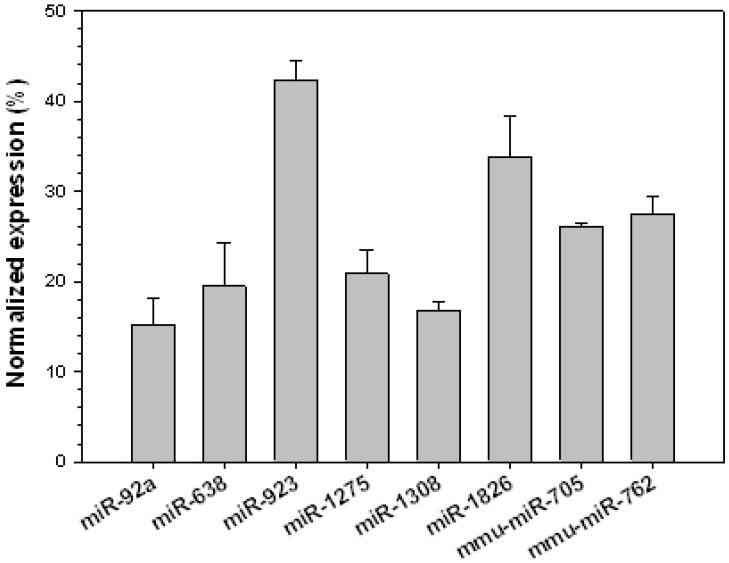
Novel miRNAs up-regulated in rabbit VX2 tumors. Signal intensities of each miRNA were calculated after normalizing the expression levels with positive controls. Data are presented as mean ± SD of triplicate samples.

In this study, we report the expression of six miRNAs that were not described previously in human liver cancers (Figure 3). Among these miRNAs, the functions and target genes for miR-923, miR-1275, and miR-1308 are unknown. It would be interesting to see what, if any, role they play in human cancers. miR-92a was suggested to target p21 in various cancer cell lines [45], and miR-1826 was shown as a prognostic biomarker for colorectal cancer [46], whereas miR-638 promotes melanoma metastasis and protects these cells from autophagy and apoptosis [47]. miR-638 was also shown to cause differentiation and proliferation of leukemic cells by targeting cyclin-dependent kinase 2 (CDK2) [48], and inhibition of cell proliferation by targeting phospholipase D1 in gastric cancer [49]. In addition to these miRNAs, we also found elevated expression of two miRNAs, miR-125b and miR-26a, that were shown previously to play important roles in organ development and cellular differentiation, respectively.

The most abundant miRNA in the liver is miR-122. miR-122 was originally identified as a critical factor that modulates the replication of hepatitis virus in the human liver [50]. Interestingly, it is down-regulated in rodent and human HCC [51]. In this study, we found that, in addition to lowered levels of miR-122, expression of miR-223, miR-34a, and miR-127 was also down-regulated. miR-223 is noteworthy because it is frequently suppressed in human HCC [52], and miR-34a, miR-127 and miR-200b are down-regulated in the rat liver during experimental hepatocarcinogenesis [36]. Unlike these other tumors, we found that miR-200b is up-regulated in the rabbit VX2 tumor.

## 4. Conclusions

MicroRNA profiles have been reported in the literature for lung, liver, kidney, breast, esophageal, thyroid and colon cancer [4,32,39,53,54,55,56,57]. Our interest in the rabbit VX2 tumor model stems from the fact that it is a frequently used animal model for human HCC [15,58,59]. When compared with the profiles obtained from patient samples reported in the literature, we found very little commonality with the rabbit VX2 profile. Although certain miRNAs expressed in each of these cancers were seen in VX2 tumors, there is no reasonable match with any single cancer profile. This could be due to the fact that the original VX2 tumor was a virus-induced anaplastic squamous cell carcinoma, and has been propagated by numerous serial transplantations into different tissues over the years. As a result, it is possible that the tumor has acquired characteristics of different kinds of tumors. However, biology of the tumor does respond to the environment of the tissue where it was implanted, as shown recently in a metabolic study of the liver tissue [60]. Given the significant disparities observed, these findings suggest that caution is warranted when data from rabbit VX2 experiments are obtained and the findings are extrapolated to human cancer therapies.
